# Impact of the COVID-19 pandemic on Self-Harm and self-harm/suicide Ideation and subsequent mortality in Northern Ireland: a longitudinal, population-wide data linkage study.

**DOI:** 10.23889/ijpds.v7i3.1899

**Published:** 2022-08-25

**Authors:** Aideen Maguire, Lisa Kent, Denise O'Hagan, Euan Paterson, Dermot O'Reilly

**Affiliations:** 1 Queen's University Belfast; 2 Northern Ireland Public Health Agency

## Objectives

To utilise Northern Ireland’s unique population-wide registry of self-harm and self-harm/suicide ideation, linked to other administrative health and death records, to examine the impact of the COVID-19 pandemic and related restrictions on presentations for self-harm/ideation and subsequent mortality risk.

## Approach

Longitudinal, population-wide registry data on all presentations to Emergency Departments for self-harm (SH) or self-harm/suicide ideation (I) (Apr 2102-2020) were linked to primary care registration and death records within Northern Ireland Trust Research Environment (NITRE). Data were divided pre-restrictions (Apr 2012-Feb 2020) and during-restrictions (Mar 2020-Sept 2020). Raw pre- and during numbers were compared. Auto Regressive Integrated Moving Average (ARIMA) models were trained considering trends and seasonal effects. Monthly forecasts were compared to actual numbers, at population level and within sociodemographic groups (gender, age, method). Survival analysis examined risk all-cause/suicide mortality after presenting with SH/I before versus during restrictions.

## Results

Mar and Apr 2020 saw 15.3% and 32.9% drop respectively in the total number of presentations for SH/I. From May 2020 numbers returned to within 2% of the monthly average. The sharp decrease in Mar/Apr 2020 was observed in both males and females but varied by age sub-categories. Presentations in those aged >64years remained as expected and presentations in the <16 years were higher than expected, compared to a decrease in presentations in those aged 16-64years. The restrictions impacted on variations by method of harm and by hospital. Suicide mortality given previous SH/I appears to increase slightly for males during the COVID restrictions with further analysis underway.

## Conclusion

Although at a population level it looks like Emergency Department presentations for SH/I decreased in first 2 months of the pandemic before returning to “normal”, the presentation profile changed dramatically by age, method and hospital. Further ARIMA predictive modelling and survival analysis are underway to explore subsequent mortality risk.

